# Geometrical Distribution of *Cryptococcus neoformans* Mediates Flower-Like Biofilm Development

**DOI:** 10.3389/fmicb.2017.02534

**Published:** 2017-12-19

**Authors:** William Lopes, Mendeli H. Vainstein, Glauber R. De Sousa Araujo, Susana Frases, Charley C. Staats, Rita M. C. de Almeida, Augusto Schrank, Lívia Kmetzsch, Marilene H. Vainstein

**Affiliations:** ^1^Centro de Biotecnologia, Universidade Federal do Rio Grande do Sul, Porto Alegre, Brazil; ^2^Departamento de Física, Instituto de Física, Universidade Federal do Rio Grande do Sul, Porto Alegre, Brazil; ^3^Physics of Living Systems, Department of Physics, Massachusetts Institute of Technology, Cambridge, MA, United States; ^4^Instituto de Biofísica Carlos Chagas Filho, Universidade Federal do Rio de Janeiro, Rio de Janeiro, Brazil; ^5^Instituto Nacional de Ciência e Tecnologia – Sistemas Complexos, Universidade Federal do Rio Grande do Sul, Porto Alegre, Brazil

**Keywords:** biofilm, cryptococcus, scanning electron microscopy, geometrical, fungi

## Abstract

Microbial biofilms are highly structured and dynamic communities in which phenotypic diversification allows microorganisms to adapt to different environments under distinct conditions. The environmentally ubiquitous pathogen *Cryptococcus neoformans* colonizes many niches of the human body and implanted medical devices in the form of biofilms, an important virulence factor. A new approach was used to characterize the underlying geometrical distribution of *C. neoformans* cells during the adhesion stage of biofilm formation. Geometrical aspects of adhered cells were calculated from the Delaunay triangulation and Voronoi diagram obtained from scanning electron microscopy images (SEM). A correlation between increased biofilm formation and higher ordering of the underlying cell distribution was found. Mature biofilm aggregates were analyzed by applying an adapted protocol developed for ultrastructure visualization of cryptococcal cells by SEM. Flower-like clusters consisting of cells embedded in a dense layer of extracellular matrix were observed as well as distinct levels of spatial organization: adhered cells, clusters of cells and community of clusters. The results add insights into yeast motility during the dispersion stage of biofilm formation. This study highlights the importance of cellular organization for biofilm growth and presents a novel application of the geometrical method of analysis.

## Introduction

Microorganisms have been traditionally analyzed using planktonic microbial cells; however, this lifestyle is not necessarily related with the growth of microbes in their most prevalent habitat. Recent approaches in confocal microscopy and molecular biology have provided evidence that biofilm formation represents the most common mode of microbial growth in nature (Costerton et al., 1995; Jabra-Rizk et al., 2004; Ramage et al., 2009; Martinez and Casadevall, 2015) and is a response to ecological competition in the environment (Oliveira et al., 2015). A wide range of microorganisms are able to switch from a planktonic to a colonial lifestyle in the form of a biofilm, creating aggregated communities that are enclosed by an extracellular matrix (ECM) (Costerton et al., 1995).

Microbial biofilms are now recognized as highly structured and dynamic communities, in which phenotypic diversification allows microorganisms to adapt to diverse environments under different conditions (Watnick and Kolter, 2000; Parsek and Fuqua, 2004; Drescher et al., 2016; Gulati and Nobile, 2016; Sheppard and Howell, 2016). Importantly, biofilms can be composed of thousands of cells encased in a matrix and attached to a surface, but they can also contain as few as tens of cells arranged as small clusters or aggregates (Stacy et al., 2016). Open channels interspersing the microcolonies allow water and nutrients to reach their interior and contribute to the nutrition and formation of mature biofilms, possibly mimicking a primitive circulatory system. Waste products might also be removed through this system (Flemming and Wingender, 2010).

Cells growing within biofilms exhibit unique phenotypic features compared to their planktonic counterparts, with the increased resistance to antimicrobial agents provided by biofilms being the more drastic example (Martinez and Casadevall, 2006a; Clatworthy et al., 2007; Lewis, 2008; Ramage et al., 2009). Biofilm formation in the environment and in the host can be induced by sub-lethal concentrations of antibiotics or secondary metabolites, respectively (Kumar and Ting, 2013; Oliveira et al., 2015). In this context, biofilm formation is an important feature of *Cryptococcus neoformans* because it is an environmentally ubiquitous fungal pathogen that causes cryptococcosis, a lethal disease with a worldwide distribution related to bioclimatic conditions as well as to soil characteristics and land use (Cogliati et al., 2017). Almost 200,000 deaths per year are estimated to be due to cryptococcal meningitis (Rajasingham et al., 2017). The major virulence factor of this fungus is the polysaccharide capsule that surrounds the cell wall and is responsible for fungal attachment to surfaces and subsequent biofilm formation (Martinez et al., 2010; de S Araújo et al., 2016). The *C. neoformans* capsule is composed mainly of glucuronoxylomannan (GXM), a polysaccharide generated intracellularly and exported to the extracellular space via vesicle-mediate secretion (Rodrigues et al., 2007). GXM is also a constituent of the cryptococcal biofilm ECM (Martinez and Casadevall, 2005; Park et al., 2009).

*C. neoforma*ns can form biofilms on medical devices, including ventriculoatrial shunt catheters (used to manage intracranial hypertension), peritoneal dialysis fistulae, cardiac valves and prosthetic joints (Walsh et al., 1986; Braun et al., 1994; Banerjee et al., 1997; Johannsson and Callaghan, 2009; Shah et al., 2015). On biotic surfaces, after traversing the blood brain barrier in meningoencephalitis, *C. neoformans* has the ability to form biofilm-like structures known as cryptococcomas (Aslanyan et al., 2017).

Although previous studies using confocal microscopy provided initial insights into cryptococcal biofilm structure, conventional scanning electron microscopy (SEM) techniques do not preserve the mature biofilm ultrastructure (Martinez and Casadevall, 2005, 2007). The highly hydrated matrix is greatly deformed and the cell samples undergo distortion and may present artifacts. Also, *C. neoformans* capsule is sensitive to dehydration and is easily disrupted during routine sample preparation (Edwards et al., 1967; Sakaguchi, 1993). As a consequence, considerable effort is currently being spent on the development of new methods and instrumentation for its visualization. By applying an adapted protocol for SEM, we characterized the underlying geometrical structure of cell distribution during biofilm formation. The degree of order was numerically quantified and we revealed a correlation between higher levels of biofilm formation and more ordered underlying structures. Order/disorder are very relevant in physical systems. In crystals, for example, deformations can only occur near defects due to the high energetic cost of their occurrence elsewhere. Besides, some phase transitions are defect mediated. Moreover, in the last decades the interplay among defects, geometry and statistical physics has been highlighted (Nelson, 2002). Here we propose the application of parameters designed to measure order in physical systems (Nelson and Halperin, 1979; Aeppli and Bruinsma, 1984; Okabe et al., 2000; Bernard and Krauth, 2011; Borba et al., 2013) to the microbial populations. We also investigated the details of the ultrastructural organization of cryptococcal biofilms and show that cryptococcal cells aggregate with a specific ordered structure favoring biofilm formation as compared to disorganized conglomerates.

## Materials and methods

### Microorganisms

*C. neoformans* var. *neoformans* B3501 strain, serotype D (ATCC 34873), is a strong biofilm former on different surfaces (Martinez and Casadevall, 2005). This serotype has an increased risk of infections for patients with skin lesions (Dromer et al., 1996). *C. neoformans* var. *grubii* strain H99 serotype A (ATCC 208821) is responsible for the vast majority of central nervous system infections, particularly in HIV infected patients. The acapsular *cap67* mutant was obtained by chemical mutagenesis of B3501 (Fromtling et al., 1982). *C. neoformans* var. *grubii* strain H99 was employed as a recipient for creating the *grasp* hypocapsular mutant involved in unconventional protein secretion (Kmetzsch et al., 2011). All of the strains were kept frozen in glycerol and subcultured at the time of the experiment. Standard biosecurity safety procedures been carried out according to our institution guidelines (www.cbiot.ufrgs.br/index.php/manual/).

### Quantification of *Cryptococcus neoformans* biofilm formation by XTT

Cells were grown for 24 h at 30°C, in 25 ml of *Sabouraud* broth media in a rotary shaker at 150 rpm. Then, the cells were collected by centrifugation at 3,000 g for 5 min, washed three times with phosphate-buffered saline pH 7.2 (PBS), counted using a hemacytometer and suspended at 10^7^ cells/ml in DMEM—Dubelcco's modified eagle media high glucose (GIBCO, USA) at pH 7.4. After that, 500 μl of the suspension were added into individual wells of polystyrene 24-well plates (Greiner Bio-One, AUS) containing sterile glass coverslips and incubated at 37°C for 48 h. Following incubation, wells were washed in triplicate with PBS to remove any planktonic cells. Then, 300 μl of XTT salt solution (1 mg/ml in PBS) and 24 μl of menadione solution (1 mM in acetone; Sigma-Aldrich) were added to each well. Microtiter plates were incubated at 37°C for 5 h. Mitochondrial dehydrogenases in live cells reduce XTT tetrazolium salt to XTT formazan, resulting in a colorimetric change, which was measured in a microtiter reader at 492 nm (SpectraMax i3). Microtiter wells containing only culture media but no *C. neoformans* cells were used as negative controls.

### Scanning electron microscopy preparation

An improved protocol developed for visualization of *C. neoformans* planktonic cells by electron microscopy was recently described (de S Araújo et al., 2016). Here, we modified a few parameters in order to preserve the ultrastructure of the biofilm stages. Briefly, after the incubation period (4 h for adhesion or 48 h for mature biofilm) as previously described, the wells containing the coverslips were washed three times with PBS. After washing, cryptococcal adhered cells were fixed with 500 μl of 2.5% glutaraldehyde type 1 (Sigma Aldrich, USA) diluted in 0.1 M sodium cacodylate buffer pH 7.2 and for 15 min at room temperature. Then, the wells were washed three times in 0.1 M sodium cacodylate buffer pH 7.2 containing 0.2 M sucrose and 2 mM MgCl_2_ with the aid of two pipettors, which were used for addition and concurrent removal to avoid air exposure. Adhered cells were dehydrated in a series of freshly made solutions of graded ethanol (30, 50 and 70%, for 5 min/step, then 95% and twice 100%, for 10 min/step). The dehydration was closely monitored to prevent biofilm matrix and capsule polysaccharide extraction. Samples were then subjected to critical point drying (EM CPD 300, Leica) immediately after dehydration, mounted on metallic stubs, sputter-coated with a 15–20 nm gold-palladium layer and visualized in a scanning electron microscope (Carl Zeiss EVO® MA10 or EVO® −50 HV Carl, Oberkochen, Germany), operating at 10 kV. Microscopic fields were selected by random scanning and photo documented. The experiment was performed in three independent replicates.

### Time-lapse microscopy

*C. neoformans* B3501 cells were prepared as described above and suspended at 10^6^ cells/ml in DMEM. After that, 1 ml of the suspension was added into individual wells of glass and incubated for 4 h at 37°C. Following incubation, wells were washed in triplicate with PBS to remove planktonic cells. Then, 1 ml of DMEM was replaced and the wells incubated for 48 h at 37°C on an Espectral FV 1000 system. Time-lapse imaging was performed at 30–60 s intervals.

### Geometric analysis

The strains were allowed to grow for 4 h (adhesion stage) in the conditions described above. Following the incubation, the wells were washed three times and prepared for SEM. Images were treated with the software ImageJ (version1.48k, Java 1.8.0_65 (64-bit); National Institutes of Health, USA, [http://imagej.nih.gov/ij]) to extract information for further statistical analysis. An ellipse was fitted to each of the particles in the image and its area was calculated. Since only the center coordinates of each ellipse are necessary for the analysis of the neighboring cells network, we represent particles with circles of fixed radius such that their areas are equal to the average ellipse area. Once the centers are defined, it is possible to establish the nearest neighbors of each cell *n*_*i*_ by means of a Delaunay triangulation and Voronoi tessellation using Fortune's algorithm (Fortune, 1987) with the software voronoi (version 1, Steve J. Fortune, Bell Laboratories, USA [http://ect.bell-labs.com/who/sjf/voronoi.tar]) With this information, it is possible to obtain the number of nearest neighbors and distance distributions, allowing one to calculate average parameters that quantify order in the spatial distribution of cells for each sample image.

To calculate the local variance in the number of neighbors $\mu_{2}^{i}$, first the average number of neighbors was calculated for the sample

(1) $$n=\langle n_{i}\rangle =\frac{1}{N}\sum_{i=1}^{N}n_{i},$$

where *N* is the number of cells whose distance to any image border is not within 5% of the system size. Cells close to the border were not taken into account to calculate bulk properties. The local variance was defined as

(2) $$\mu_{2}^{i}={\left(n_{i}-n\right)}^{2},$$

and the global variance was defined as its average

(3) $$\mu_{2}=\mu_{2}^{i}=\frac{1}{N}\sum_{i=1}^{N}\mu_{2}^{i}.$$

**Figure 1:**
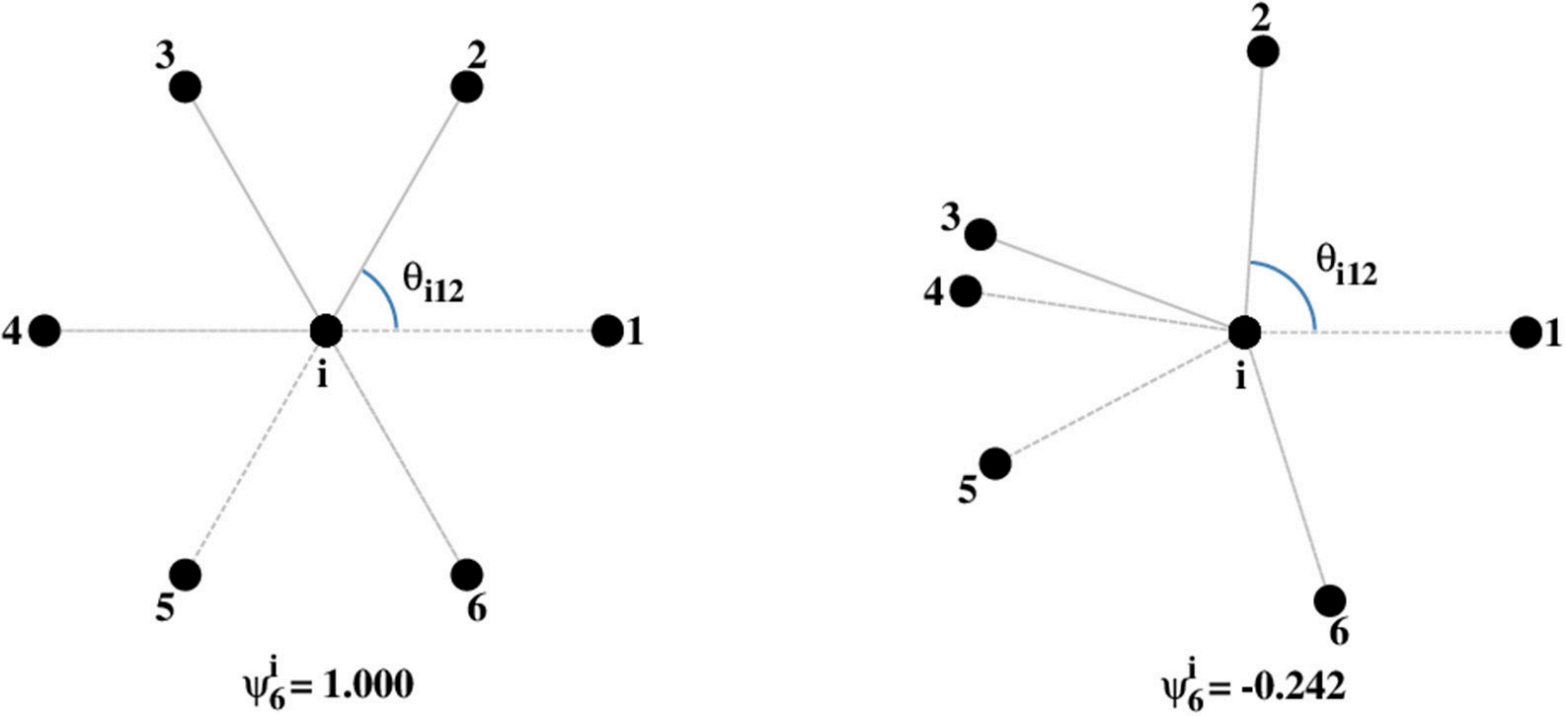
Values of $\psi_{6}^{i}$ for a site in a triangular lattice (**left**) and for a random distribution of neighbors (**right**).

Following Borba et al. (2013) we calculate the local order parameter

(4) $$\psi_{6}^{i}=\frac{1}{n_{i}}\sum_{j=1}^{n_{i}}cos\left(6\theta_{ijk}\right),$$

where θ_*ijk*_ are the angles formed by the two line segments joining site *i* and two of its consecutive neighbors (Figure 1). A given set of points (an image) can then be characterized by the average value of this quantity

(5) $$\psi_{6}=\langle \psi_{6}^{i}\rangle =\frac{1}{N}\sum_{i=1}^{N}\psi_{6}^{i}.$$

For a triangular lattice of points, every site will have $\psi_{6}^{i}=1,$ since every site has six neighbors and six angles equal to 60°. Therefore, ψ_6_ = 1 characterizes a perfect hexagonal symmetry (Borba et al., 2013). On the other hand, for a set of randomly distributed points, a value of ψ_6_ close to 0 is expected.

In order to analyze the probability of generating a given value of ψ_6_ from a random distribution of points, we generated 1,000 sets of randomly distributed non-overlapping disks with areas equal to the average area of a cell in a box of the same size as that of the image. For such an ensemble with *N*_*e*_ images (sets of points), one can calculate the average of ψ_6_ and its standard deviation. Then, it is possible to apply a Student-*t* test to evaluate the possibility that the values of ψ_6_ for experimental configurations of cells present the same distribution as those for a random configuration generated by the deposition of non-overlapping disks. The one-tailed test for samples with unequal variance was calculated using LibreOffice (version 4.2.8.2 Build ID: 420m0 (Build: 2); LibreOffice, The Document Foundation, [http://www.libreoffice.org]). The *p*-values of the test were obtained for the 3 pairs of data: (a) 5 samples of B3501 with 1,000 sets of non-overlapping disks (*p* ≈ 3.7 × 10^−7^); (b) 5 samples of H99 with 1,000 sets of non-overlapping disks (*p* ≈ 0.35); (c) 5 samples of B3501 with 5 samples of H99 (*p* ≈ 1.1 × 10^−6^). In Figure S1 we show the corresponding network for a set of randomly distributed non-overlapping disks and the distribution of ψ_6_ for 1,000 random sets.

## Results

### Biofilm formation and analysis of cellular adhesion geometry

**Figure 2:**
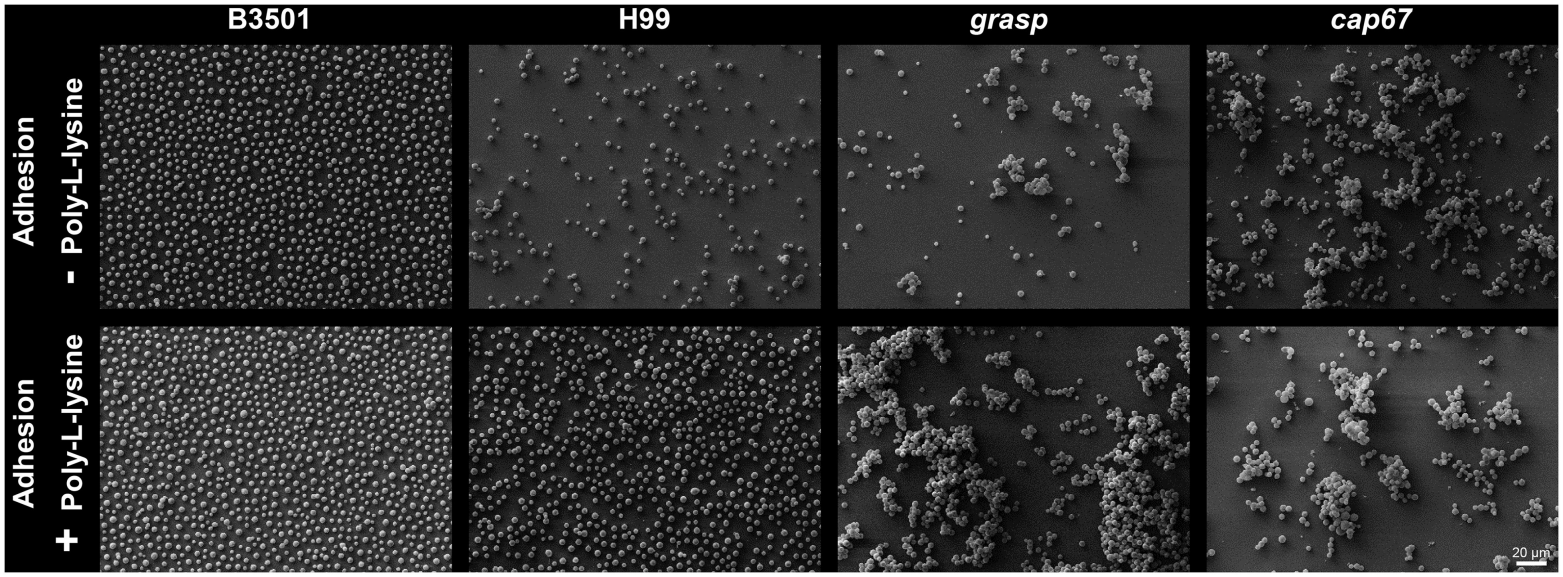
Geometric distribution of cells in the initial steps of biofilm formation, after incubation for 4 h. *C. neoformans* B3501, H99, hypocapsular *grasp* mutant and acapsular *cap67* mutant strains adhered in non-covered glass coverslips **(Top)** and PLL covered glass coverslips **(Bottom)**.

To characterize the underlying geometric structure of cell distribution in the initial steps of biofilm formation (after incubation for 4 h), SEM images were examined. This analysis considered the wild type strains *C. neoformans* B3501 and H99, the hypocapsular *grasp* mutant (Kmetzsch et al., 2011) and the acapsular *cap67* mutant (Fromtling et al., 1982). To take into account differences in adhesion of the cells to the substrate, we analyzed conditions differing only in the presence or not of poly-L-lysine (PLL). Pre-treatment with PLL increases the number of adhered cells, as expected, but did not influence the cell organization (Figure 2).

To classify the spatial distribution of cells, we used four measures: average number of nearest neighbors *n* and its variance μ_2_, intercellular distances, and the average degree of hexagonal order ψ_6_. This last measure has been applied to study the liquid-hexatic transition (Nelson and Halperin, 1979; Bernard and Krauth, 2011) and also to the study of nanoporous alumina arrays in which case the ordering and organization are crucial for engineering applications (Borba et al., 2013). Among the parameters used, ψ_6_ was the one with more conclusive results (Table S1).

**Figure 3:**
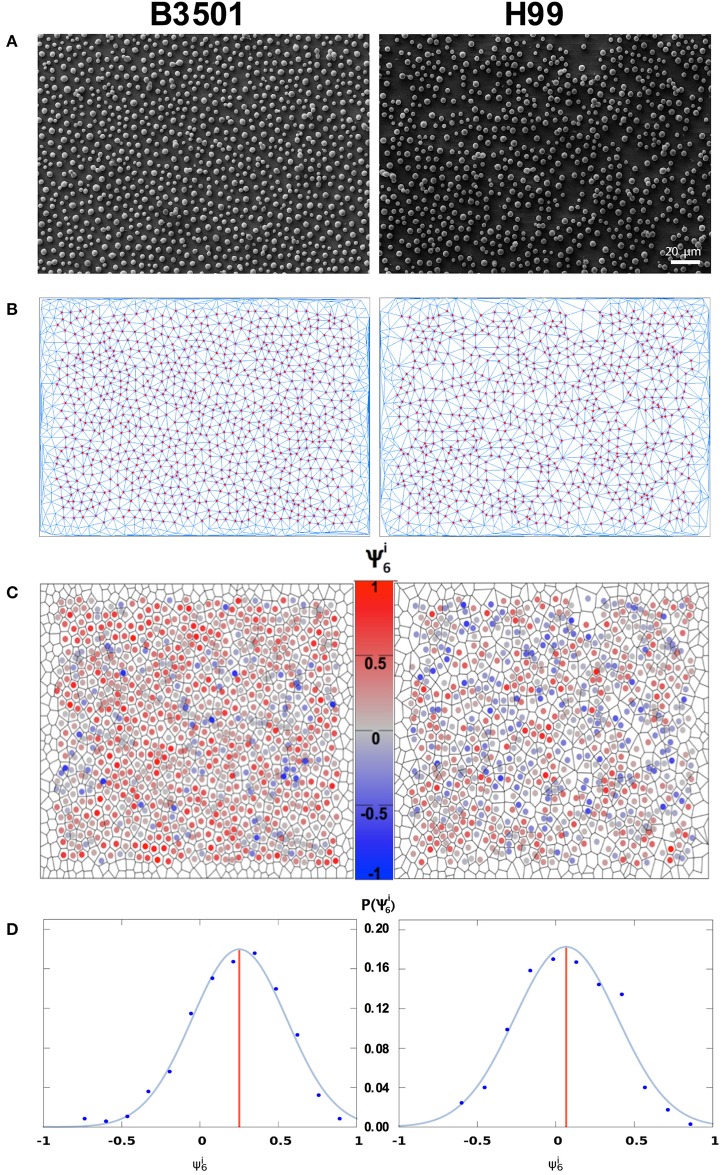
Analysis of the local order parameter, $\psi_{6}^{i}$, for samples with poly-L-lysine. Left column *C. neoformans* B3501. Right column *C. neoformans* H99. **(A)** SEM image of cryptococcal cells after 4 h of adhesion. **(B)** Network of adhered cells; nearest neighbors were obtained by Delaunay triangulation (blue line segments). Red circles represent cell centers in the bulk of the sample. Vertices without circles near the borders represent cells that were discarded from the analysis (distance to the border within 5% of system size) to minimize border effects. **(C)** Voronoi diagram of the sample. The color code represents $\psi_{6}^{i}$. **(D)** Distribution of $\psi_{6}^{i}$. The blue curve is a Gaussian fit to the data points, grouped into bins. The red vertical line displays the average value ψ_6_ of the local order parameter $\psi_{6}^{i}$ (Left: ψ_6_ = 0.22, total number of cells in the image *N*_*tot*_ = 1066, number of analyzed cells *N* = 837, average number of neighbors *n* = 6.01. Right: ψ_6_ = 0.06, *N*_*tot*_ = 885, *N* = 699, *n* = 6.01).

The variance of the number of nearest neighbors μ_2_ (Equation 2) was found to be low, in the range 0.7–2.1, indicating that the majority of cells have a number of nearest neighbors very close or equal to the average *n* = 6 (see Figure S2 for the local values $\mu_{2}^{i}$). This fact reinforces that it is adequate to use ψ_6_, a measure appropriate for hexagonal symmetry. For its quantification, we first measure the local order parameter $\psi_{6}^{i}$ (Equation 4), that indicates how isotropically arranged the nearest neighbors of the i^th^ cell are. If the cells are found in a perfect triangular lattice, all sites have six nearest neighbors and the lines joining the i^th^ cell with two of its consecutive neighbors form an angle of 60°. In this case of perfect hexagonal symmetry, the average of $\psi_{6}^{i}$ over the whole sample will attain its maximum value ψ_6_ = 1. For a random distribution of cells, with angles differing from one another, the local values $\psi_{6}^{i}$ will be smaller and their average, ψ_6_, will be negative and close to zero (Figure 1). In the case of partial ordering, one finds intermediate values 0 < ψ_6_ < 1. In Figures 3A,B, we present the Delaunay triangulation (Okabe et al., 2000), that determines the network of nearest neighbors, combined with SEM of the adhesion stage of biofilm formation with PLL biofunctionalization for B3501 and H99 (wild types). Acapsular *cap67* mutant and hypocapsular *grasp* mutant were not analyzed since they organize in 3D aggregates.

In Figure 3C, cell color is related to $\psi_{6}^{i}$. It also shows the Voronoi diagram (Okabe et al., 2000), which separates the figure into polygons such that every point in a polygon is closer to the cell inside it than to any other cell. The distribution of $\psi_{6}^{i}$ for each image is presented in Figure 3D with a Gaussian fit to the data. The red vertical line represents the average value ψ_6_.

**Figure 4:**
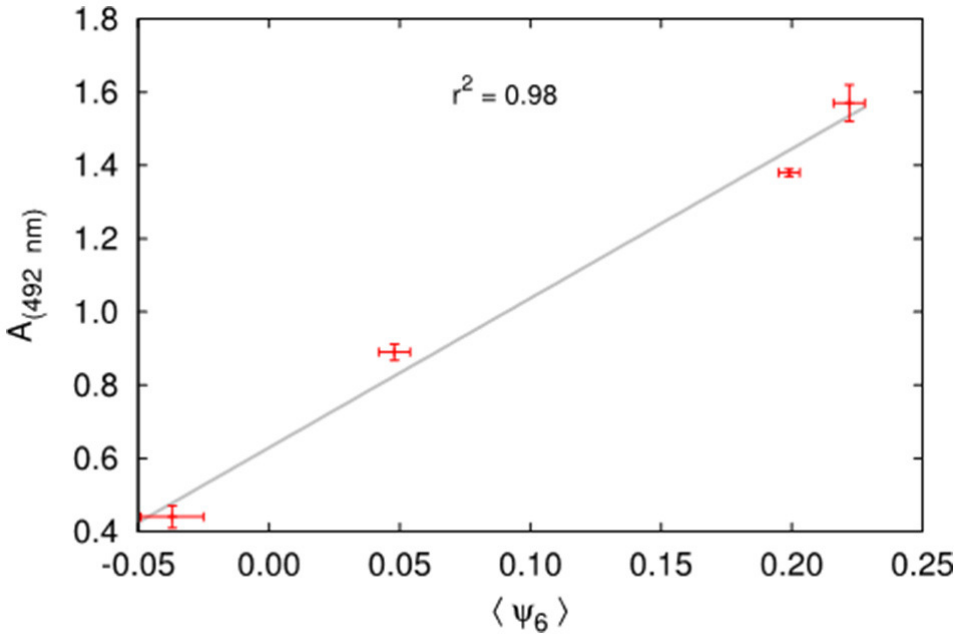
Correlation between biofilm formation measured by the XTT reduction assay (A _(492__nm)_) and the order parameter ψ_6_ for the two strains with and without PLL.

**Figure 5:**
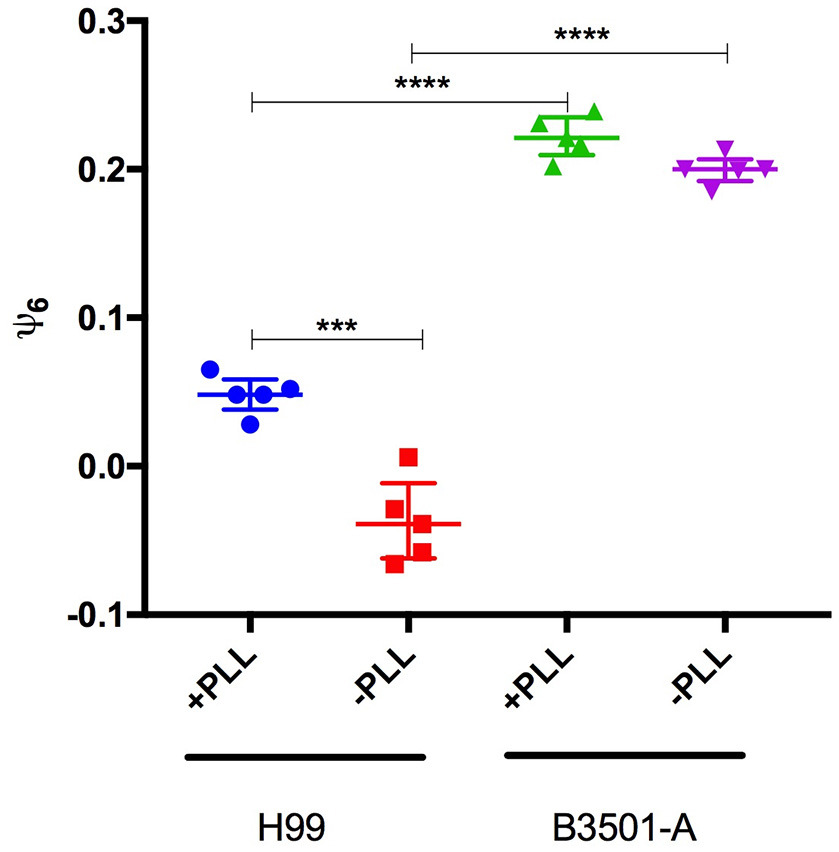
Poly-L-lysin influence on ψ_6_ is strain dependent. ψ_6_ is determined for five independent images of *C. neoformans* H99 and B3501 incubated in plates treated or not with Poly-L-Lysin. Data is presented as symbols and are also represented by the Median and inter-quartile ranges. Statistically significant differences were marked (^***^p < 0.001; ^****^p < 0.0001) according to ANOVA test followed by Tukeys multiple comparison test.

The quantification of biofilm formed after 48 h of incubation was based on 2,3-bis(2-methoxy-4-nitro-5-sulfophenyl)-5-[(phenylamino)carbonyl]-2H-tetrazolium-hydroxide (XTT) reduction assay measurements which determines the absorbance (A _(492__nm)_) of metabolic activity and correlates with biofilm formation and fungal cell number. We found a correlation (*r*^2^ = 0.98, Figure 4) between higher levels of biofilm formation [A _(492__nm)_] and more orderly underlying structures < ψ_6_ >, where the angled brackets represent an average over 5 images in the early phase of biofilm formation, since the first layer of cells on a substrate is not necessarily characteristic of a random deposition. For instance, *C. neoformans* strain B3501, known as a strong biofilm producer has < ψ_6_ > = 0.20 and A _(492__nm)_ = 1.38, while strain H99, a weak biofilm producer, has < ψ_6_ > = −0.04 and A _(492__nm)_ = 0.44 without PLL (Figure 5).

Special attention should be given to these two strains in the presence of PLL; the better-known biofilm producer B3501 has a significantly more orderly disposal than H99, with an approximately equal number of cells (Figure 2). These results endorse that the difference in biofilm formation is not due simply to different numbers of cells due to the low adhesion of H99 in the absence of PLL. For both mutants *cap67* and *grasp*, it was not possible to calculate ψ_6_ due to the formation of cell agglomerates that result in 3D structures. Nevertheless, this does not weaken the conclusion that a non-random disposal with a regular distance and number of neighbors between cells is important for biofilm formation, given that the cells are closely packed together in these agglomerates.

High values of ψ_6_ for *C. neoformans* B3501 in the range 0.18–0.21 (without PLL, Table S1) for the 5 biological replicates analyzed are indeed representative of a non-random distribution since a Student-*t* test to evaluate the possibility that such values arise from a random deposition of non-overlapping disks with the same average area as the cells yields a *p*-value *p* ≈ 3.7 × 10^−7^ (Figure S1). For the five samples of *C. neoformans* H99, we obtained *p* ≈ 0.35 when comparing its ψ_6_ values to those of the randomly deposited non-overlapping disks. The same test applied comparing *C. neoformans* B3501 with *C. neoformans* H99 yielded *p* ≈ 1.1 × 10^−6^. Therefore, it is plausible to conclude that the *C. neoformans* H99 samples present an essentially random distribution, whereas *C. neoformans* B3501 do not (Table 1).

**Table 1:** Values of ψ_6_ for 5 samples of each of the strains B3501 and H99 (without PLL).

**Biological replicate**	ψ_**6**_ **values of** ***C. neoformans*** **strains**	
	**B3501**	**H99**
1	0.213	−0.058
2	0.200	−0.029
3	0.200	−0.058
4	0.185	−0.039
5	0.199	0.006

**Figure 6:**
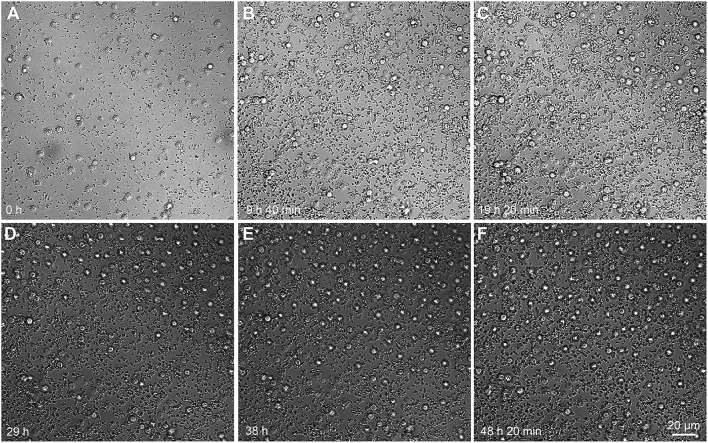
Time-lapse microscopy of *C. neoformans* B3501 flow during various stages of biofilm formation. Initial steps of biofilm formation **(A–C)**. From 24 h onwards, cells enter the vision field from the top **(D)** and auto-organize **(E–F)**. Cells tend to follow similar trajectories. Snapshots from the Movie S1.

The orderly distribution of cells even during the detachment stage of biofilm is a process that occurs dynamically (Figure 6, Movie S1). From the snapshots, it can be seen that new cells that flow into the already populated region do so by following almost the same paths and tend to maintain a more or less regular distance from other cells. This suggests that the ordering stems from some interaction between the cells: this behavior is similar to equally charged spheres (not in electrolyte solutions), which tend to auto-organize due to the electrical repulsion among themselves (Figure 5). The nature of such interaction, however, remains a point of further investigation (since the typical distance between the cells is considerably larger than the Debye length of an electrolyte solution as the cellular medium). On the other hand, our results also suggest that this effective repulsion may depend on the capsule integrity since both mutants with capsular defects tend to agglomerate into compact clusters of cells.

### *C. neoformans* biofilms are organized in flower-like clusters

**Figure 7:**
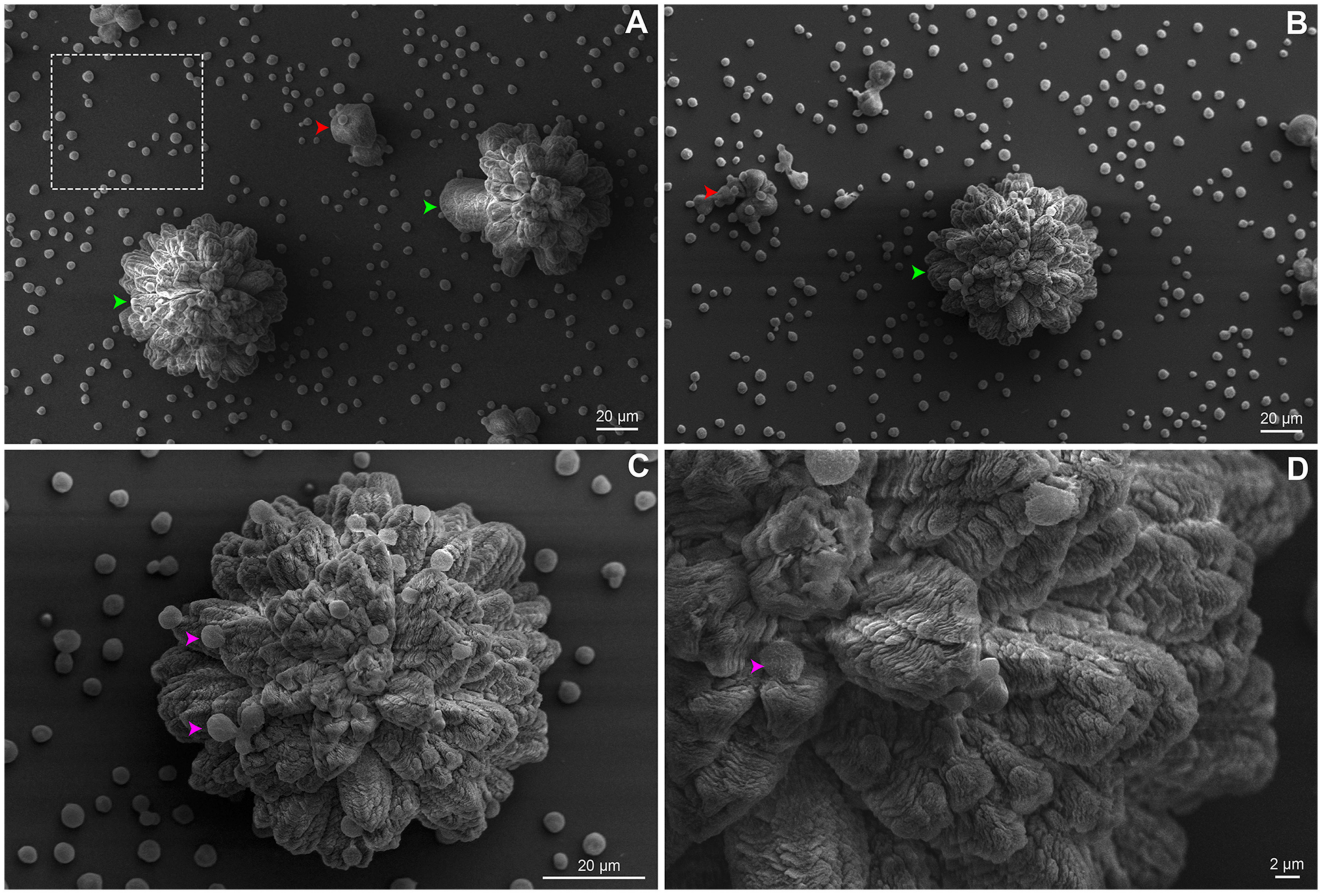
SEM of *C. neoformans* B3501 displaying flower-like clusters. Biofilm presented complex structure and spatial organization. **(A)** The dotted square indicates cryptococcal cells attached to the surface. **(A,B)** Biofilm clusters with amorphous and asymmetrical structure (red arrows) and mature biofilm with flower-like shapes (green arrows). Flower-like cluster shown in higher magnification **(C,D)** with embedded cells in the ECM (pink arrows).

We believe that the organized geometrical structure is an important factor for the next steps of biofilm formation. The strain B3501 was used for ultrastructure analysis of biofilm due to its significantly more orderly disposal during adhesion stage (Figure 2) and strong biofilm formation (Martinez and Casadevall, 2007). The applied protocol allowed the detailed observation of preserved cryptococcal biofilms by SEM. Our findings contrast with available data of confocal and optical microscopy in which resolution and detail are limited, but converge in biofilm thickness in the range of 50–76 μm, as well its complexity (Martinez and Casadevall, 2006a,b, 2007; Robertson and Casadevall, 2009). The images showed the ECM embedding cells organized into biofilm clusters with both amorphous and organized flower-like structures of the mature biofilm (Figures 7A–D). The vertical growth seems to dominate cluster expansion with regions of high ECM densities. Isolated yeast cells were found both attached to the surface and cluster-associated (Figures 7A,C,D).

**Figure 8:**
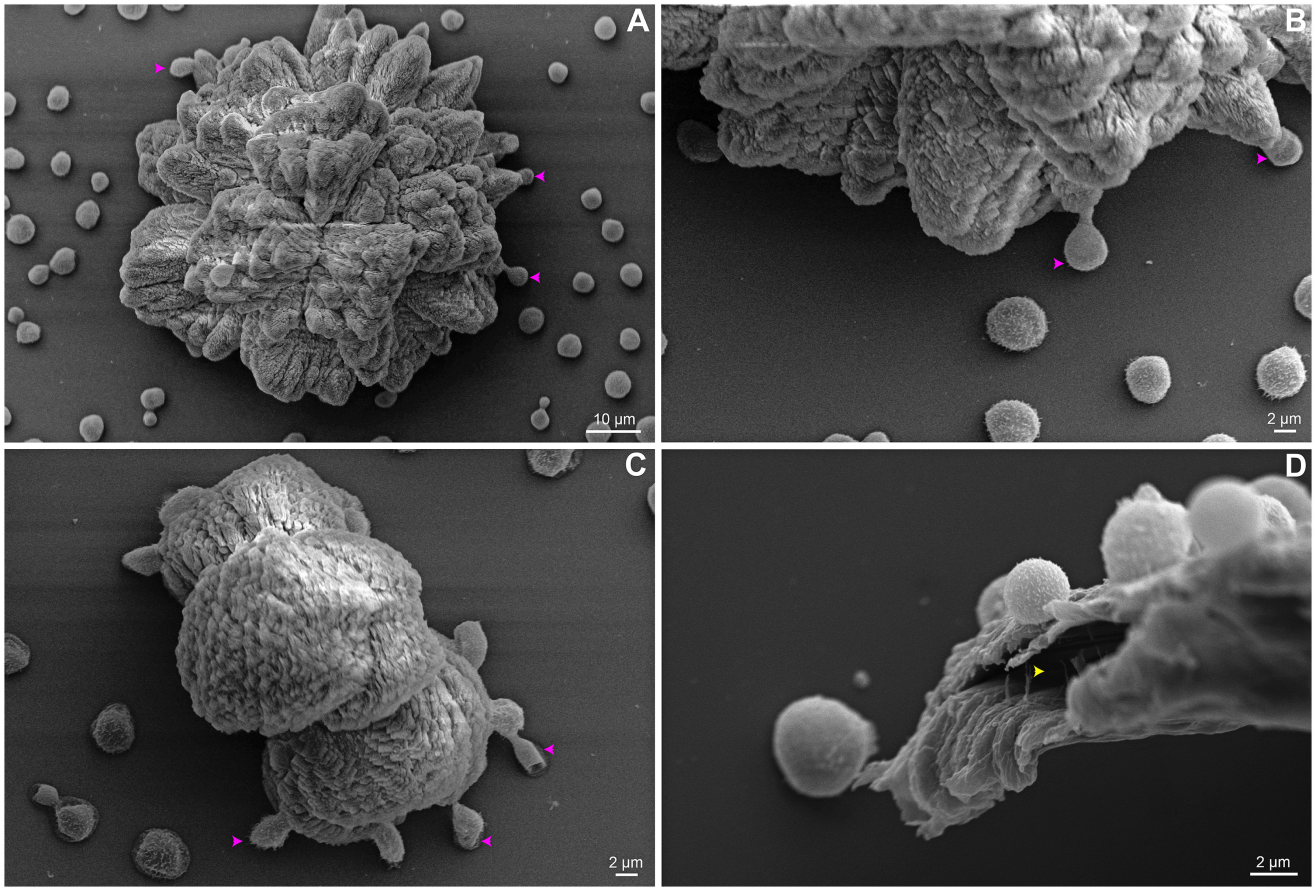
SEM of *C. neoformans* B3501 showing phenotypic cell change and biofilm channel. Anchoring cells (pink arrows) are observed located at the base of some flower-like **(A,B)** and amorphous **(C)** clusters boundaries. Micro channels (yellow arrow) are found associated with cells **(D)**.

Interestingly, an unexpected phenotype of a few cells located at cluster boundaries, resembling bridges and involved in anchoring the clusters, can be observed. These cells are elongated and interconnect surface substrate and clusters (Figures 8A–C). We found ECM micro channels displaying a well-designed structure associated with cryptococcal cells (Figure 8D). For some soil bacteria, the presence of ECM micro channels are required for cell alignment and advancement on surfaces (Berleman et al., 2016).

### Cryptococcal biofilms form a social community of clusters

Even within a community composed of genetically identical microbial cells, there exists high heterogeneity in morphology and physiology of its sub-populations (Lidstrom and Konopka, 2010). Wang et al. (2013), discovered that *C. neoformans* responds in a paracrine manner to a secreted protein responsible for colony communication and morphology (Wang et al., 2013).

**Figure 9:**
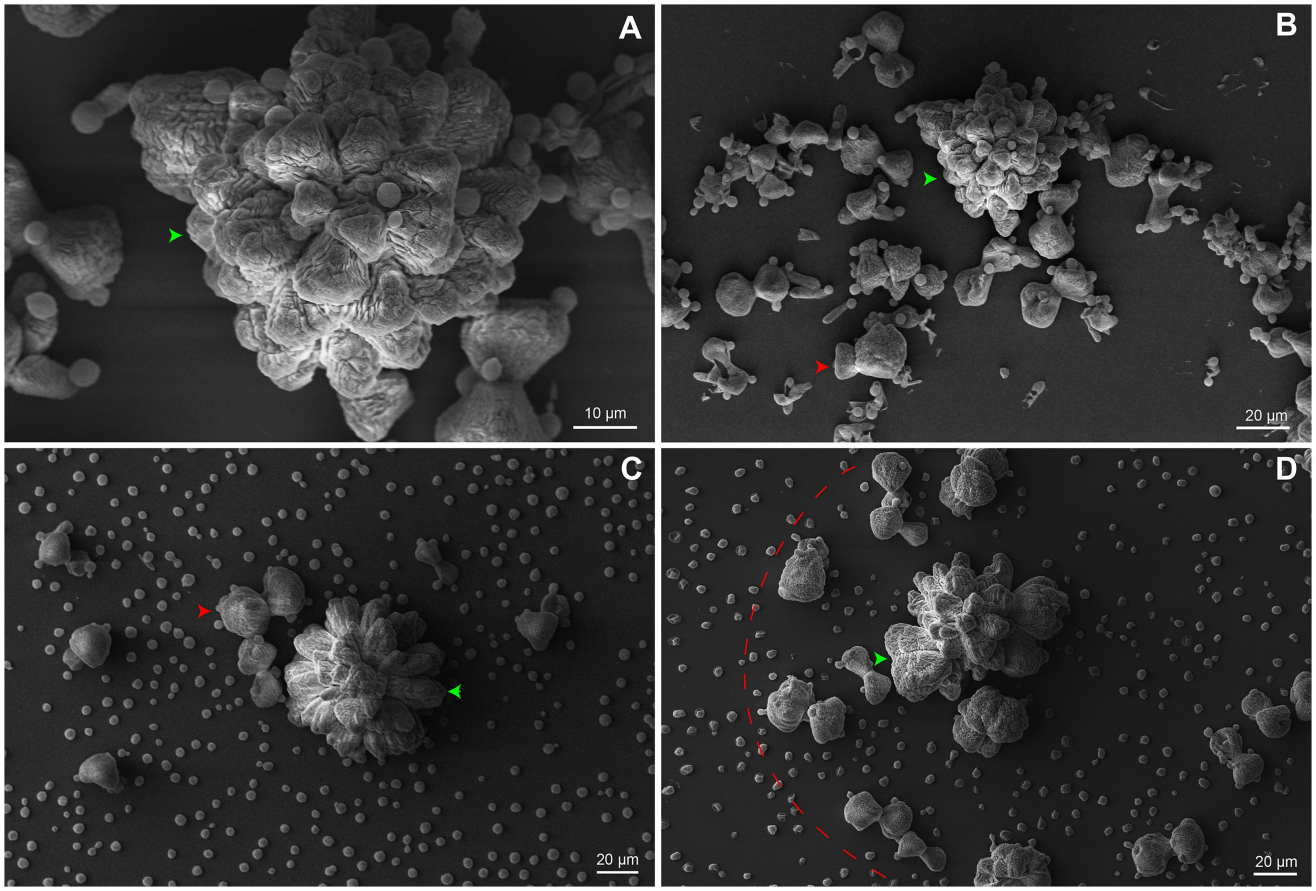
SEM of *C. neoformans* B3501 organized in a community of clusters. **(A)** Higher magnification image of a mature biofilm. **(B–D)** Small amorphous clusters (red arrows and dotted arc) surround the mature biofilm (green arrow).

To synchronize social microbial behavior, extracellular signals must disseminate across the community and reach adjacent cells. Here, we speculate the existence of a hierarchical biofilm organization composed of a cluster community. The SEM images show that small clusters (Figures 9B–D) are adjacent to the mature biofilm cluster (Figure 9A). We hypothesize that a feedback response of mature clusters signaling leads to the formation of small aggregates surrounded by ECM. The clustering process may implement a secondary signaling for functional or phenotypic switch in a paracrine manner, as supported by Wang et al. (2013). This process seems to trigger an autoinducer activity by stimulating neighboring cells to phenocopy the mature cluster.

## Discussion

The hallmarks of this study were the use of a numerical measure to quantify the geometrical order of the first layer of adhered cells in the process of biofilm formation as well as the detection of well-shaped ultrastructure of *C. neoformans* biofilms. To verify the relation between cellular order and biofilm production, we analyzed *C. neoformans* H99 and the mutants *grasp* and *cap67*, and the usual model *C. neoformans* B3501. Once we showed that there is indeed a correlation between increased order and increased biofilm production, we focused on the standard strain to further study the ultrastructure.

This analysis was made possible by the introduction of a modified protocol for SEM visualization of microbial biofilms, due to the fact that standard protocols greatly distort the matrix. To minimize artifacts, a shortened time of fixation and careful dehydration is optimal for ultrastructural SEM analysis (Joubert et al., 2015). The ultrastructure preservation was achieved by combining appropriated techniques, a reduced period of incubation during SEM preparation and good grade reagents.

Distinct levels of spatial organization were observed: adhered cells, clusters of cells, as well as the community of clusters. The affinity of attachment to different surfaces is strongly related to the presence of the cryptococcal capsule. In fixed *C. neoformans* cells, the fibers surrounding the cell (capsule filaments) directly stretch and link cells to surface, promoting attachment (de S Araújo et al., 2016).

Cryptococcal biofilm formation seems to be driven by a communication system via adhesion/matrix protein signaling (Wang et al., 2013) and directional proliferation of the original adhered cells. Cfl1, the first prominent ECM secreted protein of *C. neoformans*, is highly expressed in subpopulations located at the periphery of a mating community and is concentrated in the extracellular matrix boundary. This protein orchestrates yeast-hypha morphotype transition, cell adhesion, and virulence. This suggests that Cfl1 possibly serves as a signal regulating morphotype transition in the cells enclosed or adjacent to the ECM (Wang et al., 2012, 2013; Wang and Lin, 2015).

We hypothesize that the reversible cell attachment is mediated by capsule interactions and the orderly distribution of cells, as described above. As the capsule is primarily responsible for the high negative zeta potential of *C. neoformans* cells, variations in the structure of GXM could also influence the Zeta potential. However, zeta potential determinations of *C. neoformans* H99 and B3501 strains did not reveal major differences (Kozel and Gotschlich, 1982; Nosanchuk and Casadevall, 1997; Cordero et al., 2011). In this way, we assume that biofilm formation capability is a serotype-dependent process and is influenced by either biological or environmental factors. Based on this, the resulting patterns observed for H99 and B3501 strains cannot be explained by charge distribution. The ordering reported is due to an effective repulsion among cells, the nature of which remains a mystery. It may be due to chemical sensing or excluded volume that hinders the free motion of cells (Movie S1), in which cells flow following similar paths and tend to adhere at an approximately constant distance from one another.

**Figure 10:**
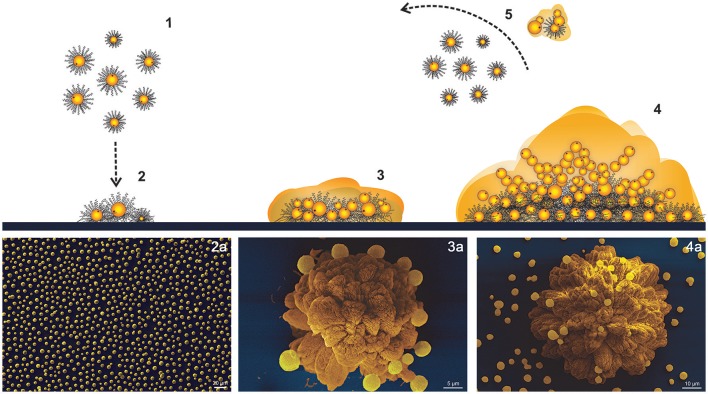
Scheme of *C. neoformans* B3501 biofilm formation. (1,2) Adhesion of planktonic cells follows an approximately hexagonal distribution. (3) Cluster expansion and shaping. (4) Flower-like mature biofilm. (5) Detachment of microcolonies or planktonic cells. (a) SEM of biofilm development stages.

Our data supports that once irreversible attachment occurs, cryptococcal cells may form a narrow ECM layer around the cell body where cells rapidly proliferate, but the surface-attached and peripheral anchored cryptococcal cells may restrict their expansion to the plane. As initial small clusters proliferate, their shape increasingly becomes anisotropic. At this point, the biofilm consists of several layers of cells grouped into clusters resembling extremely organized flower-like patterns. After maturation, cells may detach as microcolonies or as isolated planktonic cells, which auto-organize following an approximately hexagonal distribution. Cells tend to follow similar trajectories and may initiate the process again (Figure 10 and Movie S1).

Yan et al. (2016) discovered that the cluster ultrastructure of *Vibrio cholerae* biofilm results from the combination of expansion and confinement of surface-attached cells that generates an effective anisotropic stress. Such stress overpowers the cell-to-surface adhesion force for cells at the cluster center, causing these cells to realign in the vertical direction and forcing the transition from 2D expansion to 3D growth (Yan et al., 2016). Moreover, if selection pressure is high, it has been shown that clusters of *Pseudomonas aeruginosa* have higher fitness than isolated cells because cells at the top of the clusters have better access to nutrients (Kragh et al., 2016). Cluster morphogenesis results from a great number of variables capable of shaping the ultrastructure. Physical and demographic processes are demonstrated to act as key factors in biofilm architectures (Hödl et al., 2014). Cryptococcal cells are likely most susceptible to the hydrodynamics constraints due to low motility. In contrast, more motile microorganisms may escape these constraints and develop biofilm morphogenesis related to cellular migration and biofilm coalescence (Hödl et al., 2014).

Mathematical studies have related that biofilm architecture depends on the availability of nutrients, carbon and oxygen, uptake processes linked to hydrodynamics and diffusion limitation of substrate transport through the biofilm. More generally, metabolic capabilities, genotypic and phenotypic adaptations could result in different behaviors within the biofilm, allowing organisms to choose between a number of strategies (Klapper and Szomolay, 2011; Klapper, 2012).

Interestingly, for *V. cholerae* the presence of low cell number in cluster biofilm results in increased volume when compared to biofilms with a larger population. The hypothesis is that the significant changes in cell–cell spacing between small and large clusters in biofilms are due to strong temporal variation in ECM composition or production levels per cell (Drescher et al., 2016). In agreement, the flower-like clusters of *C. neoformans* present a high volume of ECM and relatively low cell concentration, as supported by our findings. For instance, the shunting procedures used to treat cryptococcal meningitis hypertension are risk associated and have historically discouraged surgeons due to its complications (Liu et al., 2014; Cherian et al., 2016) since it can provide a surface for cryptococcal attachment. It is common knowledge that uropathogenic strains of *Escherichia coli* can successfully adhere to and colonize the kidney, despite the presence of high flow rates. Since kidney tubules are narrow (<50 μm), bacterial attachment patterns at even very small spatial scales can easily block them, increasing the severity of kidney infection (Melican et al., 2011).

The architectural flower-like cluster organization observed in serotype D B3501 strain might provide the yeast cells with a protected niche against antifungals, host defenses, environmental predators and dehydration. Physical differences in *C. neoformans* serotypes A and D biofilms may reflect the predilection of some serotype D strains for peripheral tissue (e.g., skin) whereas the structure of serotype A biofilms may select these strains in tissues such as the lungs (Abdulkareem et al., 2015). As demonstrated, cryptococcal cells may detach from the biofilm in an organized manner. It is plausible to assume that organization may be needed for the successful dissemination to the host. In fact, researchers showed that *Candida albicans* detached cells from biofilms are more metabolically active than planktonic cells (Uppuluri et al., 2010).

Upon this scenario, special treatment of the devices or the use of materials that hinder the initial organization may be used clinically to avoid the development of infection, by disrupting the initial organization. Moreover, the introduction of an objective measure of order (ψ_6_) obtained from an image may facilitate the analysis of whether a given surface is prone to biofilm formation.

Continued studies are required to provide a greater understanding of the importance to investigate the complications of cryptococcal meningoencephalitis associated to the spatial distribution of clusters, as well as new methods of imaging for helping the development of new anti-biofilm targets.

## Author contributions

WL and GD: did the laboratory and MEV experiments; MeV and RdA: conceived the geometrical analysis; MeV: was responsible for the calculations and implementation of the geometrical analysis; WL, MeV, SF, CS, RdA, AS, LK, and MaV: did data analysis and interpretation; WL, MeV, SF, CS, RdA, AS, LK, and MaV: conceived the experiments and wrote the manuscript.

### Conflict of interest statement

The authors declare that the research was conducted in the absence of any commercial or financial relationships that could be construed as a potential conflict of interest.
