# Supplementary material for: Geometrical Distribution of Cryptococcus neoformans Mediates Flower-Like Biofilm Development
Source: Front Microbiol. 2017 Dec 19;8:2534. doi: 10.3389/fmicb.2017.02534 (PMC5742216; doi:10.3389/fmicb.2017.02534)
Supplement: Supplementary file 1 [file Image1.PDF]

Supplementary Material

Geometrical distribution of *Cryptococcus neoformans* Mediates Flower-Like Biofilm Development

William Lopes[1#], Mendeli H. Vainstein[2#], Glauber R. de S. Araújo[3], Susana Frases[3], Charley C. Staats[1], Rita M. C. de Almeida[2, 4], Augusto Schrank[1], Lívia Kmetzsch[1], Marilene H. Vainstein[1*]

***Correspondence**

Marilene Henning Vainstein

mhv@cbiot.ufrgs.br

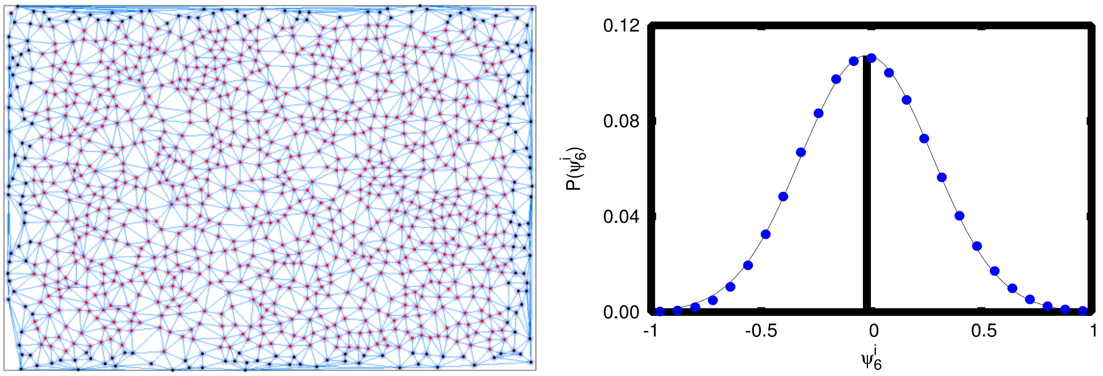

Figure S1 Set of randomly distributed non-overlapping disks and distribution of ψ_6 for 1000 random sets. A) Blue line segments compose the Delaunay triangulation network for a randomly distributed set of disks, which are not allowed to overlap (the minimum distance between 2 disks is $2R$, where the disk radius is $R \approx 7.6$ pixels, corresponding to the average cell radius of Fig. 2A). To minimize border effects, disks lying close to a boundary (a distance within 5% of the figure length) were discarded

(black dots) for the calculation of ψ_6 ($\psi_6 \approx 0.0096$, N = 867, N_{tot} = 1064). B) Distribution of ψ_6 for 1000 independent samples with the same parameters: the vertical line corresponds to the average $\langle\psi_6\rangle \approx -0.013816$. The dimensions of the box are $L_x = 1024$ and $L_y = 768$ pixels, equal to those of the image in Fig 2A.
