# Supplementary material for: Geometrical Distribution of Cryptococcus neoformans Mediates Flower-Like Biofilm Development
Source: Front Microbiol. 2017 Dec 19;8:2534. doi: 10.3389/fmicb.2017.02534 (PMC5742216; doi:10.3389/fmicb.2017.02534)
Supplement: Supplementary file 2 [file Image2.PDF]

Supplementary Material

Geometrical distribution of *Cryptococcus neoformans* Mediates Flower-Like Biofilm Development

William Lopes[1#], Mendeli H. Vainstein[2#], Glauber R. de S. Araújo[3], Susana Frases[3], Charley C. Staats[1], Rita M. C. de Almeida[2, 4], Augusto Schrank[1], Lívia Kmetzsch[1], Marilene H. Vainstein[1*]

***Correspondence**

Marilene Henning Vainstein

mhv@cbiot.ufrgs.br

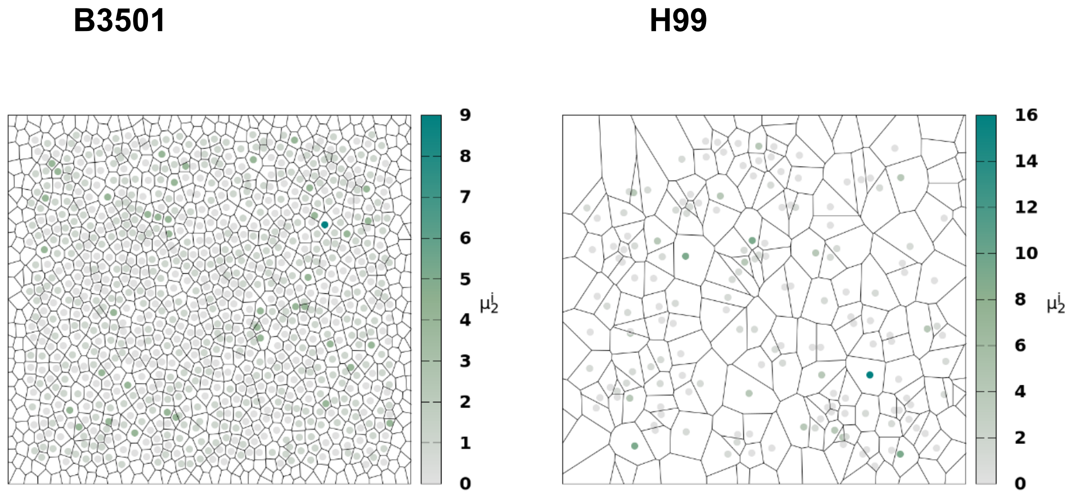

Figure S2 Analysis of the variance of the number of nearest neighbors, μ_2^i, for samples without Poly-L-lysine. Left: *C. neoformans* B3501. Right: *C. neoformans* H99.
