# Supplementary material for: Geometrical Distribution of Cryptococcus neoformans Mediates Flower-Like Biofilm Development
Source: Front Microbiol. 2017 Dec 19;8:2534. doi: 10.3389/fmicb.2017.02534 (PMC5742216; doi:10.3389/fmicb.2017.02534)
Supplement: Supplementary file 3 [file Table1.DOCX]

Supplementary Material – Table S1

**Geometrical distribution of *Cryptococcus neoformans* Mediates Flower-Like Biofilm Development**

**William Lopes^1#^, Mendeli H. Vainstein^2#^, Glauber R. de S. Araújo^3^, Susana Frases^3^, Charley C. Staats^1^, Rita M. C. de Almeida^2, 4^, Augusto Schrank^1^, Lívia Kmetzsch^1^, Marilene H. Vainstein^1*^**

***Correspondence**

Marilene Henning Vainstein

[mhv@cbiot.ufrgs.br](mailto:mhv@cbiot.ufrgs.br)

*Table S1:* Geometrical properties of 5 biological replicates of *C. neoformans* B3501 and H99, for conditions with and without poly-L-lysine.

|  | **Geometrical properties of 5 biological replicates** | | | | | | | | | | | | |  |
| --- | --- | --- | --- | --- | --- | --- | --- | --- | --- | --- | --- | --- | --- | --- |
|  |  |  |  |  |  |  |  |  |  |  |  |  |  |  |
|  | ***C. neoformans* H99 Biofilm with Poly-L-lysine (P+)** | | | | | |  |  | ***C. neoformans* H99 Biofilm without Poly-L-lysine (P-)** | | | | | |
| **sample** | **ψ6** | **μ2** | **n** | **r** | **N_tot** | **N_analized** |  | **sample** | **ψ6** | **μ2** | **n** | **r** | **N_tot** | **N_analized** |
| 2 | 0,048 | 1,09 | 6,005 | 31,8 | 944 | 740 |  | 1 | -0,066 | 1,74 | 5,898 | 66,2 | 247 | 196 |
| 3 | 0,028 | 1,52 | 5,988 | 24,0 | 1495 | 1210 |  | 2 | -0,029 | 1,62 | 5,922 | 73,4 | 213 | 166 |
| 4 | 0,052 | 1,01 | 6,005 | 33,7 | 818 | 642 |  | 3 | -0,058 | 1,64 | 5,889 | 72,6 | 209 | 171 |
| 5 | 0,062 | 1,00 | 6,007 | 32,8 | 885 | 699 |  | 4 | -0,039 | 2,09 | 5,871 | 80,4 | 179 | 140 |
| 6 | 0,048 | 0,97 | 6,006 | 33,6 | 843 | 646 |  | 5 | 0,006 | 2,01 | 5,930 | 60,5 | 272 | 215 |
| AVE | **0,048** | **1,12** | **6,002** | **31,2** | **997** | **787** |  | AVE | **-0,037** | **1,82** | **5,902** | **70,6** | **224** | **178** |
| AVE_STD | **0,006** | **0,10** | **0,004** | **1,8** | **126** | **107** |  | AVE_STD | **0,013** | **0,10** | **0,011** | **3,4** | **16** | **13** |
|  |  |  |  |  |  |  |  |  |  |  |  |  |  |  |
|  |  |  |  |  |  |  |  |  |  |  |  |  |  |  |
| ***C. neoformans* B3501 Biofilm with Polylysine-L-lysine (P+)** | | | | | | |  | ***C. neoformans* B3501 Biofilm without Polylysine-L-lysine (P-)** | | | | | | |
| **sample** | **ψ6** | **μ2** | **n** | **r** | **N_tot** | **N_analized** |  | **sample** | **ψ6** | **μ2** | **n** | **r** | **N_tot** | **N_analized** |
| 2 | 0,231 | 0,748 | 5,997 | 28,41 | 1103 | 873 |  | 1 | 0,213 | 0,667 | 6,0000 | 30,76 | 1064 | 842 |
| 3 | 0,217 | 0,828 | 6,007 | 29,20 | 1064 | 836 |  | 2 | 0,200 | 0,774 | 5,9945 | 29,67 | 1151 | 913 |
| 4 | 0,239 | 0,702 | 6,001 | 29,36 | 1032 | 810 |  | 3 | 0,200 | 0,716 | 5,9978 | 29,97 | 1127 | 894 |
| 5 | 0,221 | 0,768 | 6,001 | 29,01 | 1066 | 837 |  | 4 | 0,185 | 0,723 | 5,9988 | 30,53 | 1088 | 856 |
| 6 | 0,202 | 0,755 | 6,009 | 29,20 | 1056 | 822 |  | 6 | 0,199 | 0,687 | 6,0093 | 30,40 | 1099 | 862 |
| AVE | **0,222** | **0,760** | **6,003** | **29,04** | **1064** | **836** |  | AVE | **0,199** | **0,714** | **6,0001** | **30,27** | **1106** | **873** |
| AVE_STD | **0,006** | **0,020** | **0,002** | **0,17** | **11** | **11** |  | AVE_STD | **0,004** | **0,018** | **0,0025** | **0,20** | **15** | **13** |
|  |  |  |  |  |  |  |  |  |  |  |  |  |  |  |
| n: average number of neighbors; r: average distance between cells; N: number of cells in the image; AVE: averages; AVE_STD: standard deviations of the means | | | | | | | | | | | | | | |
|  |  |  |  |  |  |  |  |  |  |  |  |  |  |  |
|  | **Average values over 5 biological replicates** | | | | | | | | | |  |  |  |  |
|  |  | | | | | | | | | |  |  |  |  |
|  | **<ψ6>** | **std_<ψ6>** | **<μ2>** | **std_<μ2>** | **<n>** | **std_<n>** | **<r>** | **std_<r>** | **<XTT>** | **std_<XTT>** |  |  |  |  |
| **P+ H99** | **0,048** | **0,006** | **1,12** | **0,10** | **6,002** | **0,004** | **31,2** | **1,8** | **0,890** | **0,022** |  |  |  |  |
| **P+ B3501** | **0,222** | **0,006** | **0,760** | **0,020** | **6,003** | **0,002** | **29,04** | **0,17** | **1,57** | **0,05** |  |  |  |  |
| **P- H99** | **-0,037** | **0,012** | **1,82** | **0,10** | **5,902** | **0,011** | **70,6** | **3,4** | **0,44** | **0,03** |  |  |  |  |
| **P- B3501** | **0,199** | **0,004** | **0,714** | **0,018** | **6** | **0,0025** | **30,27** | **0,2** | **1,380** | **0,010** |  |  |  |  |
